# Impact of different laminae open angles on axial symptoms after expansive open-door laminoplasty

**DOI:** 10.1097/MD.0000000000011823

**Published:** 2018-08-10

**Authors:** Jizhou Wang, Tianwei Sun, Xiaoqi He

**Affiliations:** a Tianjin Medical University; b Department of Spine Surgery, Tianjin Union Medical Center, Tianjin, China.

**Keywords:** axial neck pain, cervical compression myelopathy, lamina open angle, laminoplasty

## Abstract

The present study is a retrospective study.

Axial symptoms are frequently encountered complication after laminoplasty. Some studies have reported the influencing factors and preventive measures of axial symptoms after laminoplasty. However, impact of different laminae open angles on the postoperative axial symptoms remains unclear.

The objective of the present study was to explore the effect of different laminae open angles on postoperative axial symptoms and to discuss the possible mechanisms of the impact of different open angles on axial symptoms.

We retrospectively analyzed 124 patients with multilevel cervical compression myelopathy who were treated with expansive open-door laminoplasty from February 2012 to January 2015. The operational level ranged from C3-C7 in all patients. The laminae open angles at the C4, C5, and C6 levels were measured 1 week postoperative. The mean value was taken for statistical analysis. The patients were divided into 2 groups, group A (open angles < 40°, 71 patients including 44 males and 27 females) and group B (open angles ≥ 40°, 53 patients including 32 males and 21 females). C2-C7 Cobb angle, range of cervical motion (ROM), Japanese Orthopedic Association (JOA) score, and visual analog scale (VAS) score for axial pain were compared between the 2 groups.

All patients completed at least 2-year follow-up. Both groups gained significant JOA improvement postoperatively (*P* < .05). Preoperative and postoperative C2-C7 Cobb angle and ROM comparisons were significantly different (*P* < .05) in both groups. There were no significant difference for other clinical and radiography parameters between the groups (*P* > .05). At 2 weeks and 6 months after surgery, there was significant difference in axial symptoms between the 2 groups (*P* < .05). At final follow-up, the difference between the 2 groups was not statistically significant (*P* > .05).

In different angles of the lamina open-door, incidence of axial symptoms has statistically difference between the 2 groups. When the lamina open-door angles are <40°, there are not only ensure adequate spinal cord decompression but reduces the incidence of early and midterm postoperative axial pain.

## 1 Introduction

Expansive open-door laminoplasty (EOLP), designed by Hirabayashi et al,^[1]^ has been widely used for treating multilevel cervical compression myelopathy. Some researchers have reported that EOLP is a reliable technique and results in satisfactory clinical results.^[2–5]^ However, there are still some inevitable complications after laminoplasty, including postoperative axial pain, lamina reclosure and C5 root palsy. In particular, postoperative persistent axial pain could trigger off patient's dissatisfaction after surgery, even in patients with excellent neurologic recovery.

In recent years, spinal surgeons have proposed some methods to reduce the incidence of axial symptoms, such as preserving C7 spinous process, reconstructing posterior extensor muscles, and early removal of the cervical collar.^[6–8]^ Some studies noted that the lamina open angle should be small, excessive opening angle did not reduce the incidence of reclosure but increase occurrence of complications.^[9–13]^ However, they did not continue to explore the relationship between the opening angle and axial symptoms. The objective of the present study was to explore the effect of different laminae open angles on postoperative axial symptoms and to discuss the possible mechanism of the impact of different open angles on axial symptoms.

## 2 Materials and methods

As this was a retrospective study using data routinely collected, according to a waiver issued by the ethics committee of Tianjin Union Medical Center, specific ethics approval for this study was not required.

### 2.1 Patient information

We retrospectively reviewed the data of 124 patients with multilevel cervical compression myelopathy who were treated with EOLP from February 2012 to January 2015. There were 76 men and 48 women. The age at surgery ranged from 39 to 80 years, with an average age of 63 years. The diagnosis of cervical myelopathy was confirmed both by a thorough neurologic examination and by the magnetic resonance imaging findings of spinal cord compression. The primary diseases included cervical spondylotic myelopathy (CSM) in 101 cases and ossification of the posterior longitudinal ligament (OPLL) in 23 cases. In these cases, combined radiculopathy and foraminal stenosis were included. Patients with rheumatoid arthritis, traumatic spinal cord injury, cerebral palsy, and history of cervical surgery were excluded. The operation level ranged from C3-C7 in all patients. The mean follow-up duration was 26.7 months (range 24–30 months). The operative procedure was expansive open-door using nonabsorbable sutures to secure the opened laminae.

**Figure 1:**
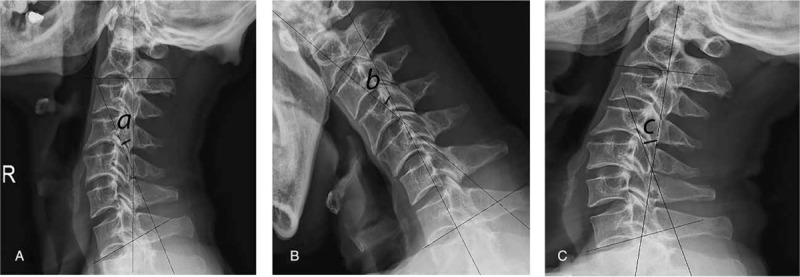
Measurement of C2-C7 Cobb angle (A), and cervical range of motion (B, C) on the lateral cervical spine X-rays. The C2-C7 Cobb angle (A) was defined as the angle of vertical line of the 2 lines, one line parallel to the inferior endplate of the C2 and the other line parallel to the inferior endplate of C7. Cervical range of motion (B, C) = difference of Cobb angle on the flexion and extension view.

In this study, laminae open angles were measured at the C4, C5, and C6 levels after laminoplasty by picture archiving and communication system software, the mean value was taken for statistical analysis. The average lamina open angle was 40.2° ± 11.3°, ranged from 20° to 69°in these cases. The patients were divided into 2 groups, group A (open angle < 40°, 71 patients including 44 males and 27 females) and group B (≥40°, 53 patients including 32 males and 21 females) according to different opening angles after surgery (Fig. 1). Neurologic status was assessed using the Japan Orthopedic Association (JOA) score before and after surgery. C2-C7 Cobb angle, range of cervical motion (ROM) were recorded before and after operation, and visual analog scale (VAS) score for axial pain (pain: VAS ≥ 3; no pain: VAS < 3) was evaluated at 1 week before surgery and 2 weeks, 6 months, and final follow-up after surgery. Early axial pain, midterm, and late axial pain after surgery were recorded, respectively. Axial pain persisting for more than 1 week during the first month after surgery was considered significant early axial pain. Axial pain persisting for more than 1 year after surgery was considered significant late axial pain. The midterm axial pain was defined as between 1 month and 1 year after surgery.

### 2.2 Surgical procedures

This surgery was performed according to Hirabayashi method.^[1]^ The patients are lying prone with the head fixed in slight flexion by a 3-pin skull fixator. A posterior midline incision was made directly above the lamina from C3-C7, followed by detachment of the bilateral paravertebral muscles from the posterior elements. Be careful to preserve the insertion of the paravertebral muscles to spinous processes of C2. Then, the C3-C7 spinous processes were removed. A high-speed drill was used to make a gutter at the medial border of the facet joint. The surgeon drilled the lamina and left a very thin cortex. The thin lamina was then opened on the open side of the lamina. A hinge-side lamina gutter was made using the same drill, but the inner cortex was not removed. The surgeon gently lifted the laminae using nonabsorbable sutures to keep each lamina open. The bone graft was put into the lateral gutters on the hinge side. The foraminotomy was performed in cases with significant radiculopathy and radiographic evidence of foraminal stenosis at the corresponding cervical level. All cases wore hard collar for 3 weeks. Exercises of the cervical muscles were started 1 week after surgery according to each patient's condition.

### 2.3 Clinical evaluation

Neurologic status was evaluated by using the JOA score and the JOA recovery rate. Recovery rate (%) = (Postoperative JOA score – Preoperative JOA score)/(17 – Preoperative JOA score) × 100%. VAS score for axial pain was recorded at preoperative 1 week, postoperative 2 weeks, 6 months, and final follow-up, respectively. The patients who experienced complications including infection, cerebrospinal fluid leakage, and lamina reclosure were recorded.

### 2.4 Radiologic evaluation

**Figure 2:**
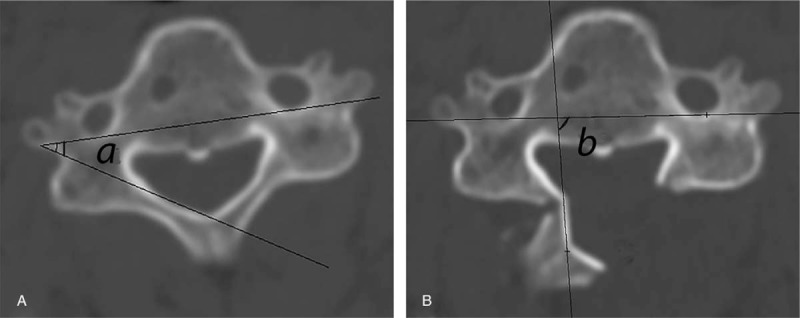
The different laminae angles were measured on computed tomography films. (A) Preoperative lamina angle a. (B) Postoperative lamina angle b. The lamina open angle = Postoperative lamina angle b − Preoperative lamina angle a.

The C2-C7 Cobb angle and ROM were measured on the lateral cervical spine X-rays at 1 week before surgery and final follow-up after surgery, respectively (Fig. 1). The different laminae angles were measured on computed tomography (CT) films at C4, C5, and C6 before and after surgery. The lamina angle was defined as the angle made by 2 crossed line, one through both sides of the vertebral body crossing transverse foramen posteriorly and the other one connecting with the inner border of the lamina in the hinge side.^[14]^ The final measurement of the lamina opening angle = postoperative angle (b)-preoperative angle (a), taking the average value for statistical analysis (Fig. 2).

### 2.5 Statistical analysis

Statistical analysis was performed using SPSS 19.0 statistical software (SPSS Inc, Chicago, IL). Continuous variables were presented as mean ± standard deviation. Independent-sample *T* tests and Chi-squared test were performed to calculate the differences between the 2 groups. The paired *t* tests were performed to detect the difference of preoperative and postoperative data. *P*-value of <.05 was defined as statistically significant.

## 3 Results

### 3.1 Clinical results

The JOA score in group A improved significantly from 9.0 ± 1.5 before the surgery to 13.7 ± 1.2 at the final follow-up, the JOA score in group B improved from 8.8 ± 1.5 before the surgery to 13.4 ± 1.4 at the final follow-up. The recovery rate was 58.7% ± 11.7% and 56.6% ± 10.8% in groups A and B, respectively. No significant difference was found in preoperative JOA scores, postoperative JOA scores, and recovery rate between group A and group B (*P* > .05). There were no significant differences between the 2 groups in sex, age, diagnosis, blood loss, operation time, duration of disease, and duration of follow-up (*P* > .05). The difference for preoperative neck pain was not significant between the 2 groups (*P* = .83) (Table 1).

**Table 1:**
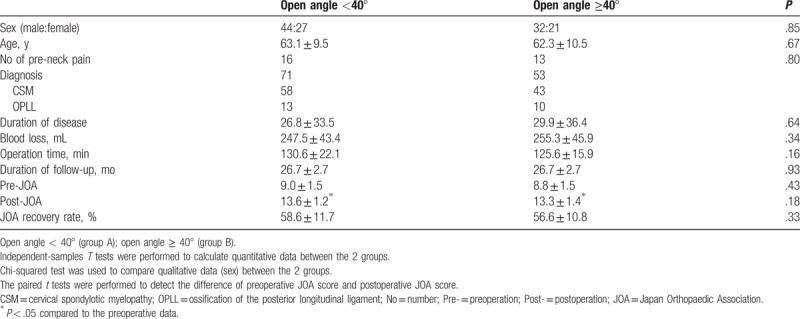
Characteristics for all patients.

### 3.2 Radiography results

**Figure 3:**
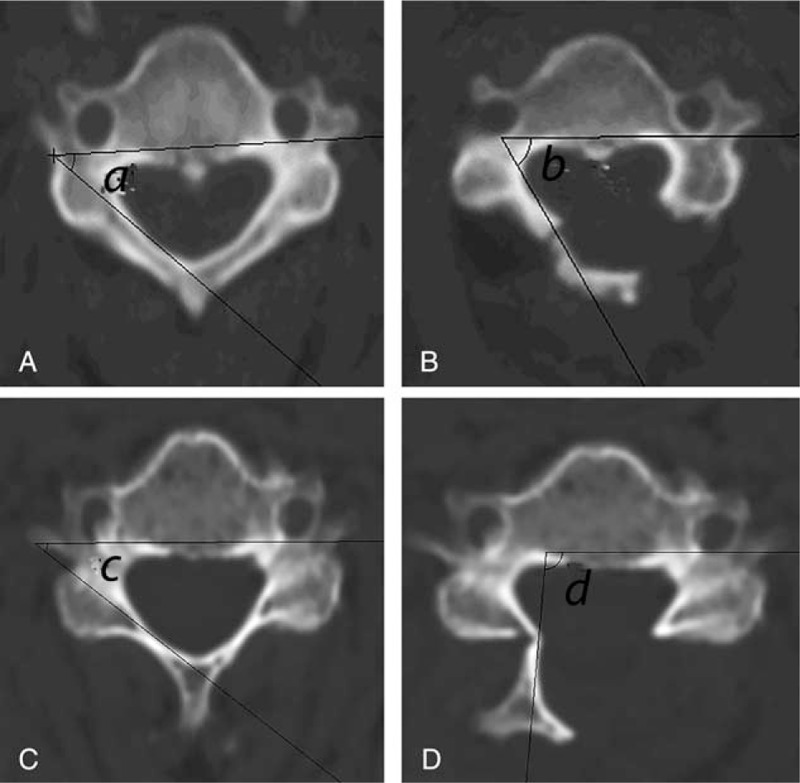
Group A: (A) Preoperative lamina angle a was 33°. (B) Postoperative lamina angle b was 64°, the lamina open angle b − a was 31°. Group B: (C) Preoperative the lamina angle c was 45°. (D) Postoperative lamina angle d was 96°. The lamina open angle d − c was 51°.

The cervical CT was reviewed a week after surgery, and the laminae angles were measured. Group A included 71 patients (open angle < 40°), and group B had a total of 53 cases (open angle ≥ 40°) (Fig. 3). Preoperative and postoperative C2-C7 Cobb angle and ROM were not statistically difference (*P* > .05) between the 2 groups. C2-C7 Cobb angle and ROM were significantly different before and after surgery for both groups. The radiography data are summarized in Table 2.

**Table 2:**
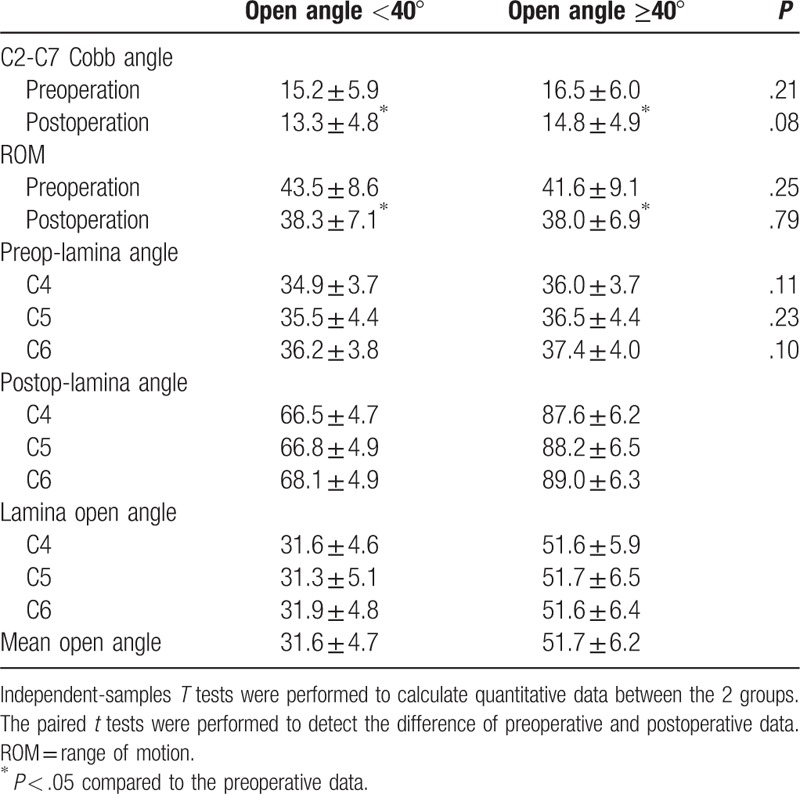
Radiography factors at postoperatively and preoperatively between the 2 groups.

### 3.3 Axial symptoms

The 10-point VAS score (pain: VAS ≥ 3; no pain: VAS < 3) for axial symptoms were examined at preoperative 1 week and postoperative 2 weeks, 6 months, and final follow-up. Preoperatively, 16 patients complained of significant neck pain in group A and 13 patients in group B. The mean preoperative neck pain VAS was 2.5 ± 2.1 and 2.4 ± 1.8 in both groups, respectively, and there was no significant difference between the 2 groups (*P* > .05). At 2 weeks after operation, there were 50 patients (21 in group A and 29 in group B, 29.6% vs 54.7%) complained of severe postoperative neck pain. The mean VAS was 2.4 ± 1.8 in group A and 3.1 ± 1.7 in the B group, the difference was significant (*P* = .03). At 6 months, there were 10 patients in group A (14.1%, VAS, 1.5 ± 1.1) and 17 patients in group B (32.1%, VAS, 2.1 ± 1.4), which was statistically significant (*P* = .03). At final follow-up, there were 3 patients in the group A (4.2%, VAS, 1.1 ± 0.80) and 5 patients in the group B (9.4%, VAS, 1.3 ± 1.1), the difference between the 2 groups was not statistically significant (*P* = .24). The axial symptoms gradually decreased in all patients during the experimental period. However, early and midterm axial pain were predominantly observed in group B (the large lamina open angle), late axial pain was no significant difference between the 2 groups. The data are summarized in Table 3. At the follow-up after 24 to 30 months, both groups did not have reclosure of the opened lamina, infection, and postoperative cerebrospinal fluid leak.

**Table 3:**
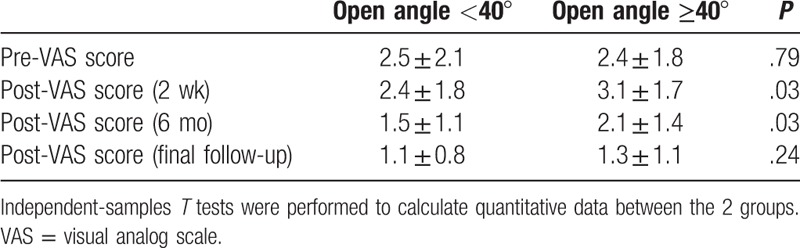
VAS scores in group A and group B preoperatively and postoperatively.

## 4 Discussion

Axial symptoms have been recognized as one of the most serious complications after posterior decompression surgery. Several possible sources have been proposed, including posterior skeletal structures destruction, facet joint damage, nerve root injury, extensor muscles atrophy, resection of C2 or C7 spinous processes, and range of cervical motion decrease.^[15–19]^

It is still unclear as to what is the appropriate open angle to obtain adequate decompression of the spinal cord. Wide opening angle is expected to reduce the risk of reclosure by changing the direction and strength of muscles attached to the lamina. However, excessive opening angle of the lamina may increase the incidence of complications. Lee et al^[10]^ noted that increase in opening angle to 40° or more did not reduce the incidence of restenosis and might be a risk factor for nerve root palsy after laminoplasty. A recent retrospective study indicated that lamina open angle of 53.5° can be an appropriate threshold between clinically adequate decompression and increasing risk of C5 palsy.^[20]^ Although their methods of measurement were different from ours, their studies showed that excessive opening angle could not acquire satisfactory clinical results. In a recent retrospective study, Zhang et al^[14]^ discussed the relationship between clinical results of EOLP with different open angles and recommended the lamina open angle should be maintained at about 30°. In that research, there was no further discussion of the effect of different lamina open angle on the incidence of midterm and late axial neck pain. In this study, when opening angles are <40°, the surgery can not only ensure adequate spinal cord decompression but minimize the impact on posterior organizational structure, thereby reducing the incidence of axial symptoms.

Our study showed that the incidence of axial pain in the large open-door group was higher than in the small open-door group. The possible reasons are as follows:

1. In single open-door laminoplasty, the spinous processes were deviated from the normal position, the large open angle correlates with more worse unsymmetry of posterior skeletal structure, resulting in obvious axial symptoms.
2. In single open–door laminoplasty, paravertebral muscles were detached from laminae, leading to atrophy of the muscles, the influence on extensor muscle strength was more prominent when lamina open angle is large.
3. The tension of suture stay in the spinous process and the facet capsule is more obvious when open angle was large, damaging the facets and soft tissue and resulting in nonbacterial inflammation around facet joint capsule.
4. Excessive opening angle cause the formation of more epidural scar tissues, compressing spinal nerve root.

The asymmetry and damage of posterior vertebral column may be one of the causes of axial symptoms. Hirabayashi et al^[21]^ noted that the biggest disadvantage of open-door laminoplasty was that the postoperative posterior skeletal structure of the cervical spine became unsymmetrical. In a prospective randomized clinical study, Okada et al^[22]^ considered that axial pain was improved in French-door laminoplasty but became worse in open-door laminoplasty, the result could be explained by the asymmetry of posterior structure after open-door laminoplasty. In a retrospective case–control study, Chen et al^[23]^ stated that the destruction of cervical posterior structures, such as the posterior muscles and bony structures might lead to axial symptoms. In our study, the large angle of opening caused more obvious asymmetry of the posterior vertebral column, leading to a higher incidence of axial symptoms.

The relationship of cervical posterior extensor muscles with persistent axial pain following laminoplasty is still controversial. In traditional open-door laminoplasty, the extensor muscles are detached from the spinous process and their insertions are damaged, leading to dystrophy and atrophy of the muscles. Some surgeons have considered the posterior muscle atrophy a source of axial pain, they found that less invasive to the posterior extensor muscles of the cervical spine was an effective procedure for preventing postoperative persisting axial pain.^[24,25]^ Kotani et al^[6]^ emphasized that employing the deep extensor muscle-preserving approach might be effective in reducing axial pain when compared to conventional open-door laminoplasty. Fujibayashi et al^[26]^ found that axial symptoms and muscle strength reduction were significantly correlated and reduction in muscle strength might easily result in the occurrence of axial pain. Some researchers hold opposite views, Hosono et al^[16]^ revealed that C7 and not the deep extensor muscles was significantly related to axial pain, extensor muscle detachment from the lamina may cause mild pain early after surgery but not result in significant chronic axial pain. Riew et al^[17]^ indicate that preservation of sub-axial deep extensor muscles plays no significant role in reducing axial neck pain after cervical laminoplasty. In this study, early and midterm axial pain between the 2 groups was statistically significant, late axial pain was no significant, the main reason might be a temporary reduction in muscle strength occurred during the early postoperative period. The muscles strength gradually recovers after 1 year. As showed in the study, Fujibayashi et al^[26]^ noted that muscle strength recovered by 3 months and had increased to 120% of the preoperative value by 12 months after the operation in most patients.

Facet capsule suture and stretch are discussed as another important factor related to axial symptoms. Wang et al^[15]^ reviewed literatures about classic laiminoplasty procedure and concluded that postoperative axial symptoms might be related to dissection around the facets and soft tissue. Chen et al^[27]^ indicated that the conventional procedure made a retention suture on the facet capsule and damaged the capsule, increasing the incidence of axial symptoms. Cohen et al^[28]^ reported that capsule stretch activates nociceptors could be a possible cause of persistent neck pain. The neurophysiologic studies considered injured facet-joint capsules as a source of the cervical spinal pain. They noted that inflammation of the facet joints leads to decreased thresholds of capsule receptors, which may then lead to persistent pain.^[29]^ In this study, the open-door was kept open by placing a suture through the facet capsule on the hinge side and through the spinous processes. When lamina open angle was large, the tension of suture in the spinous process and the facet capsule was more obvious, resulting in axial neck pain.

Stimulation and injury of the cervical nerve root branches may be associated with the occurrence of axial symptoms. Excessive opening angle created large epidural space, resulting in the formation of more epidural scar tissue to compress the nerve root. Chen et al^[27]^ concluded that the modified open-door laminoplasty using titanium miniplate was effective in preventing the incidence of axial neck pain. The possible cause was that scar tissue was prevented outside of the spinal canal by the miniplate.

Preoperative neck pain might be linked to postoperative axial symptoms. However, this problem was still an argued item. Kimura et al^[30]^ found that preoperative higher axial neck pain intensity was one of the independent predictors of postoperative moderate-to-severe axial neck pain at the 2-year follow-up. Ohnari et al^[31]^ got another result that the preoperative axial symptoms might be not related to postoperative axial symptoms. The present study showed no significant correlation between preoperative and postoperative axial symptoms.

In the present study, our patients wore a hard cervical collar for 3 weeks followed by active isometric exercise of cervical muscle. Prolonged external fixation might be more helpful in promoting hinge lateral bone healing; however, several studies have indicated that the duration of brace use is related to postoperative axial pain.^[15,19]^ The reasons may be as follows: early removal of the cervical collar and early active cervical muscle exercises might increase local blood circulation, accelerate the repair of soft tissue and prevent muscle atrophy, thereby reducing the incidence of axial symptoms.

There were several limitations in the present study. First, muscle strength was not measured before and after cervical surgery using specific measurement device. Additional study is needed to observe the relationship between changes in muscle strength and different laminae open angles. Second, the sample size of this study is small and more clinical studies are needed to explore the most appropriate open angles.

In conclusion, there is no significant difference in neurologic recovery rate between the different lamina opening angles. Incidence of axial symptoms has a statistically significant difference between the 2 groups. When laminae open-door angles are <40°, it not only ensures adequate spinal cord decompression but reduces the incidence of early and midterm postoperative axial pain.

## Acknowledgments

The authors thank all the people who give the help for this study.

## Author contributions

**Conceptualization:** Jizhou Wang, Tianwei Sun, Xiaoqi He.

**Data curation:** Jizhou Wang, Tianwei Sun, Xiaoqi He.

**Formal analysis:** Jizhou Wang, Tianwei Sun.

**Investigation:** Jizhou Wang, Xiaoqi He.

**Methodology:** Jizhou Wang, Xiaoqi He.

**Project administration:** Jizhou Wang, Tianwei Sun,

**Resources:** Tianwei Sun

**Software:** Xiaoqi He.

**Supervision:** Jizhou Wang, Tianwei

**Validation:** Jizhou Wang, Tianwei Sun, Xiaoqi He.

**Writing – original draft:** Jizhou Wang, Tianwei Sun, Xiaoqi He.

**Writing – review & editing**: Jizhou Wang, Tianwei Sun
